# Nanoscale metal–organic frameworks as photosensitizers and nanocarriers in photodynamic therapy

**DOI:** 10.3389/fchem.2022.971747

**Published:** 2022-08-26

**Authors:** Gauta Gold Matlou, Heidi Abrahamse

**Affiliations:** Laser Research Centre, Faculty of Health Sciences, University of Johannesburg, Johannesburg, South Africa

**Keywords:** nanoscale metal–organic frameworks, photodynamic therapy, photosensitizers, drug delivery system, nanocarrier and delivery, tumor therapy

## Abstract

Photodynamic therapy (PDT) is a new therapeutic system for cancer treatment that is less invasive and offers greater selectivity than chemotherapy, surgery, and radiation therapy. PDT employs irradiation light of known wavelength to excite a photosensitizer (PS) agent that undergoes photochemical reactions to release cytotoxic reactive oxygen species (ROS) that could trigger apoptosis or necrosis-induced cell death in tumor tissue. Nanoscale metal–organic frameworks (NMOFs) have unique structural advantages such as high porosity, large surface area, and tunable compositions that have attracted attention toward their use as photosensitizers or nanocarriers in PDT. They can be tailored for specific drug loading, targeting and release, hypoxia resistance, and with photoactive properties for efficient response to optical stimuli that enhance the efficacy of PDT. In this review, an overview of the basic chemistry of NMOFs, their design and use as photosensitizers in PDT, and as nanocarriers in synergistic therapies is presented. The review also discusses the morphology and size of NMOFs and their ability to improve photosensitizing properties and localize within a targeted tissue for effective and selective cancer cell death over healthy cells. Furthermore, targeting strategies that improve the overall PDT efficacy through stimulus-activated release and sub-cellular internalization are outlined with relevance to *in vitro* and *in vivo* studies from recent years.

## Introduction

Metal–organic frameworks (MOFs) are highly porous crystalline materials made up of a combination of organic ligands and metal ions or clusters (Lai et al., 2019). MOFs are applied to various fields such as catalysis, photodynamic therapy, sensing, and molecular adsorption and separations because of their extensive tunable and large surface areas, high porosity, and intriguing functionalities (Rocca and Lin, 2011; Liu et al., 2018; Lai et al., 2019). Using various synthetic methods and reaction conditions, researchers are able to significantly reduce the size of the bulk MOF down to a nanosized scale, resulting in nanoscale MOFs (NMOFs) (Gao et al., 2020). This has led to the use of NMOFs in one of two ways in biomedicine applications, as delivery agents for active agents or by incorporating the desired active agents into the matrix or simply loading them into the pores of NMOFs (Rocca and Lin, 2011). Compared to other nanocarriers, NMOFs provide multiple binding sides for high loading of active agents and high porosity for encapsulating or incorporating active agents for delivery and treatment of diseases (Gao et al., 2020).

NMOFs have been demonstrated to be applicable in immunotherapy, chemotherapy, radiotherapy, and photodynamic therapy (PDT), depending on the therapeutic agents loaded within the porous structure or linked on the binding sides (Gao et al., 2020). In PDT, a light of known and desired wavelength is directed toward a ground state photosensitizer molecule that absorbs its energy and undergoes photochemical and photophysical pathways through the excited singlet state and undergoes intersystem crossing to the excited triplet state where it transfers energy to molecular oxygen to yield reactive singlet oxygen (ROS) species or cytotoxic singlet oxygen (Chilakamarthi and Giribabu, 2017). Typically, in a tumor microenvironment, the ROS species or cytotoxic singlet oxygen are able to cause the destruction of cancer cell growth and cancer cell death (Mroz et al., 2011). NMOFs are able to meet both the specifications and abilities for use as nanocarriers, photosensitizers, or a combination of both in PDT and cancer therapy (Gao et al., 2020).

Structural properties and functions of NMOFs permit for the engineering of NMOFs with unique properties for single or multiple loading and delivery of photosensitizers, including compositional and structural tenacity, biocompatibility and biodegradability, and controlled or triggered drug release (Liu et al., 2016; Sun et al., 2019). Additionally, the nanoscale dimensions of the NMOFs allow them to easily mediate through the leaky vasculature of the tumor microenvironment to improve intracellular concentration and retention of the cargo through the enhanced permeability and retention (EPR) effect (Sun et al., 2014). One other advantage of NMOFs is their ability to be constructed with photosensitizer metal ions or organic complexes that are able to produce ROS or cytotoxic singlet oxygen upon light excitation to cause cancer cell killing (Gao et al., 2020). The presence of the metal ions in NMOFs also plays a crucial role in improving the photosensitizing properties of the drugs in the application as the metal promotes intersystem crossing of excited molecules to the excited triplet state through the heavy atom effect (Nyokong and Antunes, 2013).

Two main characteristics of NMOFs, one of which is being able to act as a photosensitizer (PS) agent and the other as a drug loading and delivery system in targeted photodynamic therapy (TPDT) are outlined in this review. We further discuss the strategies employed by various researchers during the preparation of NMOFs for application in TPDT with respect to active or passive targeting and the stimulus-responsive strategies for release at the tumor microenvironment. Most recent reviews on NMOFs in PDT have covered the modifications of NMOFs and their performance in PDT and other biomedical applications (Liu et al., 2018; Lai et al., 2019; Alves et al., 2021; Ye et al., 2022). This review outlines the structural and morphological properties of NMOFs as nanocarriers and PS agents with attention to PDT activity *in vitro* and *in vivo* and to the tumor microenvironment physiological properties. Specific modifications of NMOFs as PS agents or drug delivery systems have been developed to overcome the challenges of PDT, with some demonstrating promising effectiveness in both *in vitro* and *in vivo* applications. These modifications, the design of NMOFs, and their use in combined therapies with PDT will be summarized in this review. Lastly, the future prospects of NMOFs and the challenges to be addressed are assessed and outlined.

## Photodynamic therapy

### Photochemical and photophysical pathways

**FIGURE 1:**
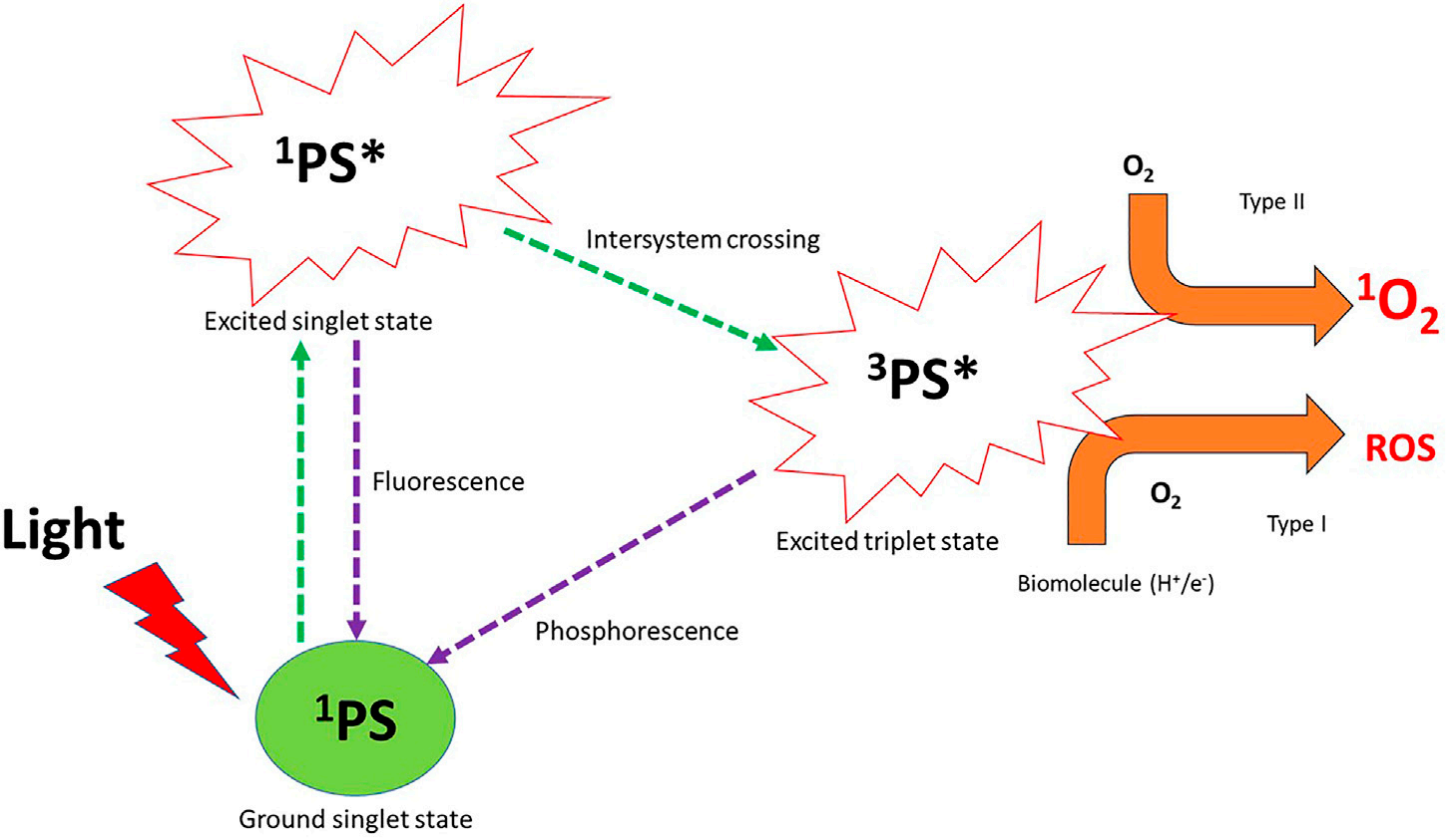
Photochemical and photophysical pathways of the photosensitizer (PS) in photodynamic therapy after laser light irradiation generate reactive oxygen species (ROS) and cytotoxic singlet oxygen. ^1^PS = photosensitizer in the ground state, ^1^PS* = photosensitizer in its excited singlet state, ^3^PS* = photosensitizer in the excited triplet state, O_2_ = molecular oxygen, ^1^O_2_ = singlet oxygen, ROS = reactive oxygen species.

Photodynamic therapy (PDT) is a non-invasive, highly effective, and selective method that is clinically approved for cancer treatment (Paszko et al., 2011). PDT is based on the generation of the cytotoxic singlet oxygen (^1^O_2_) or reactive oxygen species (ROS) that follows the photochemical and photophysical process after laser light ablation of a photosensitizer in the ground state, as shown in Figure 1. The cytotoxic singlet oxygen and ROS are able to interact with cancer cells promoting structural disintegration, unnormal functions, and subsequent death of the tumor (Chilakamarthi and Giribabu, 2017). Typically, three key components interact with each other: a light source of a desired wavelength, a photoactive and non-toxic dye, and molecular oxygen. Initially, the photoactive dye (photosensitizer (PS)) in its ground state is irradiated with the desired laser light, followed by absorption of enough energy to cause the transition of the PS agent to the excited singlet state (Figure 1). The PS agent in the excited state has a short-lived lifetime and can either undergo the desired intersystem crossing to the excited triplet state or fluorescence back to the ground state (Darwent et al., 1982). The extent of the population of the excited molecules into the excited triplet state through the intersystem crossing depends on the spin-orbit coupling (Darwent et al., 1982), which could be enhanced by the incorporation of heavy atoms into the PS structure or through conjugation with metal-based particles such as nanoparticles and NMOFs (Nyokong and Antunes, 2013).

The PS agent in the excited triplet state is able to interact with molecular oxygen to undergo one of the two pathways’ reactions or both, named Type I and Type II pathways (Figure 1). Type I reactions or pathways typically occur in the presence of biomolecules (nucleic acid, proteins, and lipids) present within the tumor tissue. The PS agent in the excited triplet state acquires a hydrogen atom or an electron within the biomolecules to generate ROS (oxidation products) such as hydroxyl radicals (HO^●^), superoxide anion (O_2_^●-^), hydrogen peroxide (H_2_O_2_), and hydroperoxide radicals (HOO^●^) (Mroz et al., 2011; Yin et al., 2015; Sun et al., 2018) (Figure 1). The ROS generated causes cell damage and obliteration of normal functions through lipid peroxidation (Mroz et al., 2011). In the type II pathway, the PS agent in the excited triplet state transfers its energy to ground state molecular oxygen to yield a highly reactive and cytotoxic singlet oxygen (Mroz et al., 2011). The cytotoxic singlet oxygen is able to interact effectively with nucleic acids, lipids, and proteins of the cell membrane to result in cell destruction which leads to cell death through apoptosis or necrosis (Mroz et al., 2011). Of the two pathways, type II is known as the most common pathway that PDT follows (Mroz et al., 2011; Calixto et al., 2016), even though both pathways can occur simultaneously; the percent availability of the oxygen molecules and the PS used and its affinity to tumor cells yield a particular balance between the pathways (Calixto et al., 2016).

### Limitations of photodynamic therapy

The efficacy of PDT on cancer cells relies heavily on the nature of the PS agent and its affinity and localization on tumor tissue. The limitations of PDT strongly rely on the nature of the PS agents over the availability of oxygen and the laser light used (Gunaydin et al., 2021). Typically, laser light with a wavelength within the therapeutic window (620–850 nm) matching the PS absorption band would be appropriate to provide sufficient energy required to activate the PS, undergo photochemical pathways, and transfer its energy to molecular oxygen to generate cytotoxic singlet oxygen or ROS (Brancaleon and Moseley, 2002). In terms of oxygen availability, the rapid growth of cancer could result in oxygen deprivation and the photosensitization process also depletes the cellular oxygen; thus, fractional light (pulse) is used to minimize the effect of oxygen deprivation during PDT (Xiao et al., 2007). One important characteristic of NMOFs is that they can be tailored with oxygen-generating abilities, a structural characteristic that is explored in relation to hypoxia stimulus-triggered NMOFs in PDT (Zhou et al., 2022).

PS agents, on the other hand, undergo various processes from accumulation within the malignant area and absorb light of correct intensity and wavelength. Therefore, the PS agent that undergoes a photodynamic reaction would require correct solubility, purity, amphiphilicity, pharmacokinetic properties, and dosimetry to achieve the desired efficacy (Gunaydin et al., 2021). In addition, since PS agents are known to have robust photoactivity in their monomeric form, they also undergo strong aggregation and lesser solubility in water which reduce their photosensitizing efficiency (Debele et al., 2015). PS agents also have limitations with accumulation, and the lack of selective localization on the malignant area at high concentrations has been demonstrated to hinder PDT efficacy (Shirasu et al., 2013). Additionally, the ROS or cytotoxic singlet oxygen with a short lifetime and limited radius within the biological system also impacts the PDT effect on the irradiated site. This makes PDT efficacy depend extensively on the targeted localization or subcellular placement of the PS agent within tumor cells or environments (Moan, 1990; Shirasu et al., 2013).

### Targeted photodynamic therapy

Targeted photodynamic therapy (TPDT) is an improved form of photodynamic therapy that utilizes a photosensitizer in the same mechanism as in PDT. A PS agent designed with cancer-targeting abilities is allowed to accumulate within the host body, where it accumulates at the disease site before being irradiated with laser light to generate cytotoxic singlet oxygen or ROS for the destruction of cancer (Darwent et al., 1982; Sobolev et al., 2000). The intention of using the PS agent with targeting abilities is to eliminate the limitations of conventional PDT or PS agents (Sobolev et al., 2000). Therefore, PS agents are commonly conjugated to cancer-targeting ligands or nanoscale particles to improve their targeting abilities and concentration within the tumor microenvironment (Ostańska et al., 2021).

Targeting pathways in TPDT, named active and passive targeting due to the modes of localizing the PS agents at the tumor microenvironment are the most common approaches used in targeting therapy of cancer (Ostańska et al., 2021). Active targeting occurs when the PS agent attached to a cancer-specific targeting ligand (antibodies, amino acids, peptides, and aptamers) directly binds to the receptors overexpressed on the surface of cancer cells (van der Meel et al., 2019). Passive targeting occurs when PS agents attached or embedded within materials such as nanoparticles or metal–organic frameworks are delivered to the tumor site through the leaky vasculature of the tumor microenvironment (Zhen et al., 2014). The major advantage of cancer-targeting PS agents is the ability to improve PS agent accumulation, specificity, and localization within the tumor microenvironment, which subsequently improve the efficacy of the short-lived cytotoxic singlet oxygen or ROS in cancer killing (Chitgupi et al., 2017). Other targeting strategies involve exploring the physiological microenvironment of the tumor to design NMOFs with photosensitizing properties for controlled release at the cancer site due to a change in pH, enzymes, oxygen availability (hypoxia), and ionic concentrations, among others (Moradi Kashkooli et al., 2020). These unique properties have led to the design of nanocarrier systems with stimuli response, controlled and specific drug release of loaded or chemically bonded drugs on NMOFs within the tumor microenvironment (Moradi Kashkooli et al., 2020). These targeting strategies of NMOFs in PDT are discussed further as follows with examples of *in vitro* and *in vivo* works that have been reported.

#### NMOFs’ active targeting in photodynamic therapy

Cancer microenvironments are known to overexpress specific receptors attached to cancer cell surfaces. These receptors are vital and serve as nutrients for the survival and aggressive growth of cancer cells (Chitgupi et al., 2017). Targeting the overexpressed receptors by incorporating ligands with affinity to the PS agent structure improves the uptake and internalization of the PS agent at the specific site by ligand–receptor binding on tumor cells (Akhtar et al., 2014). Nanoscale MOFs contain desirable porosity and surface properties to allow the attachment and encapsulation of target ligands for effective delivery, uptake, and internalization of PS agents within the cancer cells or tumor microenvironment. Ligands with high affinity to receptors overexpressed on the cancer cell surfaces such as folic acid, antibodies, peptides, proteins, and aptamers have been explored for active targeting of tumor tissue and cells (Chitgupi et al., 2017). The submicron sizes of the nanoscale MOFs also offer excellent properties to serve as efficient cargo materials as nanocarriers in drug delivery systems (DDS).

The nanosized DDS of MOFs’ backbone possesses unique properties to permeate through physiological barriers and bind to target receptors to efficiently internalize the drugs in the tumor microenvironment at high concentrations as opposed to traditionally known drugs (Parveen et al., 2012). Park et al. (2016) prepared a size-controllable Zr (IV)-porphyrin-based MOF-nanostructured DDS consisting of tetratopic linker (tetrakis (4-carboxyphenyl) porphyrin (H_2_TCPP)) and a 6-connected Zr_6_ cluster (Zr_6_(OH)_4_(H_2_O)_6_(OH)_6_(COO)_6_) denoted as PCN-224 (porous coordination network) for active targeting PDT applications on Hela and A549 cells (Park et al., 2016). The active targeting capability of the MOF-DDS was achieved by linking folic acid through a post-synthetic procedure to take advantage of the binding affinity of folic acid to folate receptors that are overexpressed on the cancer surface (Zwicke et al., 2012). The MOF-DDS with folic acid modification was found to have better PDT efficacy than the photosensitizer alone (Park et al., 2016).

In another study, Zhang et al. (2019) prepared a PCN-224 MOF-DDS for active targeting of cancer cells for both imaging chemotherapy and photodynamic therapy (Zhang et al., 2019). The nanoscale MOFs were functionalized with a carboxyl and fluorescein-modified aptamer of A549 lung cancer cells that was linked to the Zr^6+^ ions by a coordination bond and also loaded with doxorubicin (DOX) in its porous system. The DOX-PCN-224-aptamer NMOF drug system was found to localize within the cell nuclei of A549 with high specificity and affinity as compared to MCF-7 sub-cellular localization studies due to the DNA aptamer moiety of the drug system. The chemotherapeutic and PDT efficacy of the NMOF system showed improved tumor mortality after the pH-induced release of DOX facilitated apoptosis of A549 followed by photosensitizer killing of cancer cells by the porphyrin molecule of the PCN-224 MOF (Zhang et al., 2019). This demonstrates a dual targeting system where the low pH of the tumor microenvironment and the aptamer affinity was utilized to specifically localize the drug system within cell organelles.

#### NMOFs’ passive targeting in photodynamic therapy

The aggressive nature of cancer cells during their proliferation stages leads to an uncommon tissue structure and anatomy of the solid tumor (Shirasu et al., 2013). Most importantly, aggressive cell growth further leads to permeability in blood vessels, extensive angiogenesis, and an impaired lymphatic system due to inflammation and hypoxia (Mang, 2004; Shirasu et al., 2013; Attia et al., 2019). These physiological and morphological differences within the tumor microenvironment as compared to normal cells are what make up passive targeting in cancer therapy (Stuchinskaya et al., 2011; Parveen et al., 2012). Thus, passive targeting relies heavily on tumor biology (leakiness and vasculature) and drug carrier properties (circulation time, porosity, and size) (Attia et al., 2019).

Nanoscale DDS such as NMOFs are able to easily permeate through the leaky vasculature system to deliver the drug cargo through a phenomenon known as the enhanced permeability and retention (EPR) effect (Maeda et al., 2000). The size of the nanoscale DDS serves as an important characteristic during passive targeting to allow circulation, accumulation, and retention through the EPR effect in the tumor microenvironment (Chitgupi et al., 2017). Typically, nanoscale DDS with size ranges between 20 and 200 nm are considered optimal to evade both escape into the blood capillaries and clearance from the reticuloendothelial system (Kohane, 2007; Faraji and Wipf, 2009). Zheng et al. (2020) prepared an NMOF-DDS for passive targeting of cancer cells (Zheng et al., 2020) and the NMOF-DDS (MOF@POP-PEG) presented better colloidal stability and good biocompatibility due to PEGylation and controllable sizes and shape. These properties improved the NMOFs’ clathrin-mediated cellular internalization and therapeutic efficacy on Hela cells, HepG2, and U14 cervical cancer-bearing mice. The work further demonstrated the synergistic performance of both photothermal therapy and PDT of the NMOFs (Zheng et al., 2020). In another study, NMOFs with hypoxic response stimuli properties were designed recently by Yang et al. (2022) for targeting cancer cells through EPR and activated through a stimulus (hypoxia) (Yang et al., 2022). Photoactive NMOFs (UiO-AZB/HC-TPZ) with star-shaped morphology and different particle sizes were prepared, and it was discovered that particles sizes of less than 200 nm were favorable for good blood circulation and were further used in their *in vitro* and *in vivo* work (Yang et al., 2022). The importance of the size of NMOFs plays a key role in passive (EPR)-targeted therapies as demonstrated in this study as the NMOF showed high retention in the reticuloendothelial organs of tumor-bearing mice, which promoted the efficacy of cancer-killing through PDT (Yang et al., 2022).

#### NMOFs’ stimulus-triggered targeting in photodynamic therapy

The tumor microenvironment is known to possess numerous unique physiological characteristics compared to normal cells that can be explored for tunable drug release, especially in cases of loaded drugs by NMOFs (Huo et al., 2016). These physiological characteristics such as acidic (pH) microenvironment, ionic (REDOX) microenvironment, hypoxia, high levels of hydrogen peroxide, presence of hydrogen sulfide, overexpressed glutathione, and hypoxia have been demonstrated to be key features of consideration for designing cancer-targeted nanocarrier systems such as NMOFs for specific endogenous and controlled drug release in the tumor microenvironment (Huo et al., 2016; Moradi Kashkooli et al., 2020). Hydrogen sulfide (H_2_S), which is significantly high in colon adenocarcinoma cells (Hellmich and Szabo, 2015), was recently used by Ma et al. (2017) as a signaling molecule for the NMOF photosensitizer DDS (Ma et al., 2017). The NMOF-DDS was constructed from the porphyrin derivative as a bridging ligand with Cu^2+^ as metal nodes of the MOF. The release of the porphyrin photosensitizer from the DDS network was activated in the presence of H_2_S which completely breaks off the Cu^2+^ from the NMOF, thus making porphyrin to be a free monomer to be used as a PS agent drug during PDT (Ma et al., 2017). The *in vitro* and *in vivo* PDT studies of the NMOF-DDS were tested on HTC116 tumor-bearing nude mice and HepG2 and LoVo cells. High levels of H_2_S in nude mice (*in vivo*) promoted disintegration of the NMOF-DDS, improved PDT efficacy and complete removal of the tumor due to the high off-loading of porphyrin, and subsequent singlet oxygen generation as compared to low concentrations of H_2_S in HepG2 and LoVo cells (Ma et al., 2017).

Luo et al. (2019) investigated the REDOX cleavable di-(1-hydroxylundecyl) Selenium (DH-Se)/PEG/PPG as randomly polymerized coating of the PCN-224 MOF to form a poly(DH-Se/PEG/PPG urethane)@MOF shell–core nanoparticle (Luo et al., 2019). The NMOF-DDS was then loaded with DOX for use in combined chemo and photodynamic therapy on HepG2 liver cancer cells and HepG2 liver tumor-bearing nude mice. The photosensitive redox-controlled NMOF-DDS was found to highly accumulate in the intracellular environment of cancer cells and solid tumor, thus improving the efficacy of combined therapy on the cancer site (Luo et al., 2019). Another model of triggered or stimulus-responsive drug delivery and therapy that is commonly investigated is pH stimulus response due to the low acidity of the tumor microenvironment; as such, NMOFs with drug release properties that are pH responsive are designed to react to conditions such as swelling, cleavage, and protonation (Moradi Kashkooli et al., 2020).

Hypoxia, found in over 50% of solid tumors and always present in tumor growth, can provide highly diverse features to be explored for targeting and release of NMOFs in a hypoxic tumor microenvironment (Vaupel and Mayer, 2007; Patel and Sant, 2016; Moradi Kashkooli et al., 2020). The hypoxic microenvironment activates glycolysis resulting in the high production of carbonic acid (HCO^−^) and lactic acid, which is the cause of the acidic pH. The intracellular (reducing) and extracellular (oxidizing) space of the hypoxic environments also shows high redox potentials that can be explored for drug targeting (Patel and Sant, 2016). Recently, Yang et al. (2022) prepared hypoxia-responsive NMOFs consisting of an azobenzene linker (azobenzene-4.4′-dicarboxylic acid, AZB) as an organic ligand with Zr^6+^ as metal nodes (UIO-AZB). The as-prepared NMOF was further functionalized with chlorin e6 (Ce6) and a bioreductive prodrug (tirapazamine, TPZ) to the UIO-AZB/HC-TPZ NMOF-DDS for use in dual chemo and photodynamic therapy of Hela and U14 cell-bearing mice (Yang et al., 2022). The aza-group is hypoxia-sensitive, causing the disintegration of the UIO-AZB/HC-TPC NMOF-DDS in the hypoxic tumor microenvironment, thus, promoting chemotherapy by TPZ and high generation of ROS during light-triggered PDT. The combined therapy facilitated by hypoxic-triggered chemotherapy and light-triggered PDT was found to offer the highest tumor cell killing both *in vitro* and *in vivo* (Yang et al., 2022). In another study, hypoxic tumor microenvironments were targeted by Zhou et al. in their design of hypoxia-inhibiting and self-generating oxygen NMOF-DDS consisting of hypoxia-inducible factors (HIF)-1α inhibitor acriflavine (ACF), PEG, and manganese oxide (MnO_2_) that dissociate to generate Mn^2+^ and O_2_ during therapy (Zhou et al., 2022). The NMOF-DDS (ACF@PCN-222-MnO_2_-PEG (APM)) was applied for PDT on Hela cells and U14 cell-bearing female Kunming mouse models which demonstrated the best performance in both cancer killing *in vitro* and tumor suppression *in vivo* (Zhou et al., 2022). These stimulus-triggered NMOF-DDS demonstrated multifunctionality in efficient drug delivery and release promoted by a stimulus in a tumor microenvironment and were further used for selective combined therapies for effective tumor killing both *in vitro* and *in vivo*.

## Nanoscale metal–organic frameworks

### Background

**FIGURE 2:**
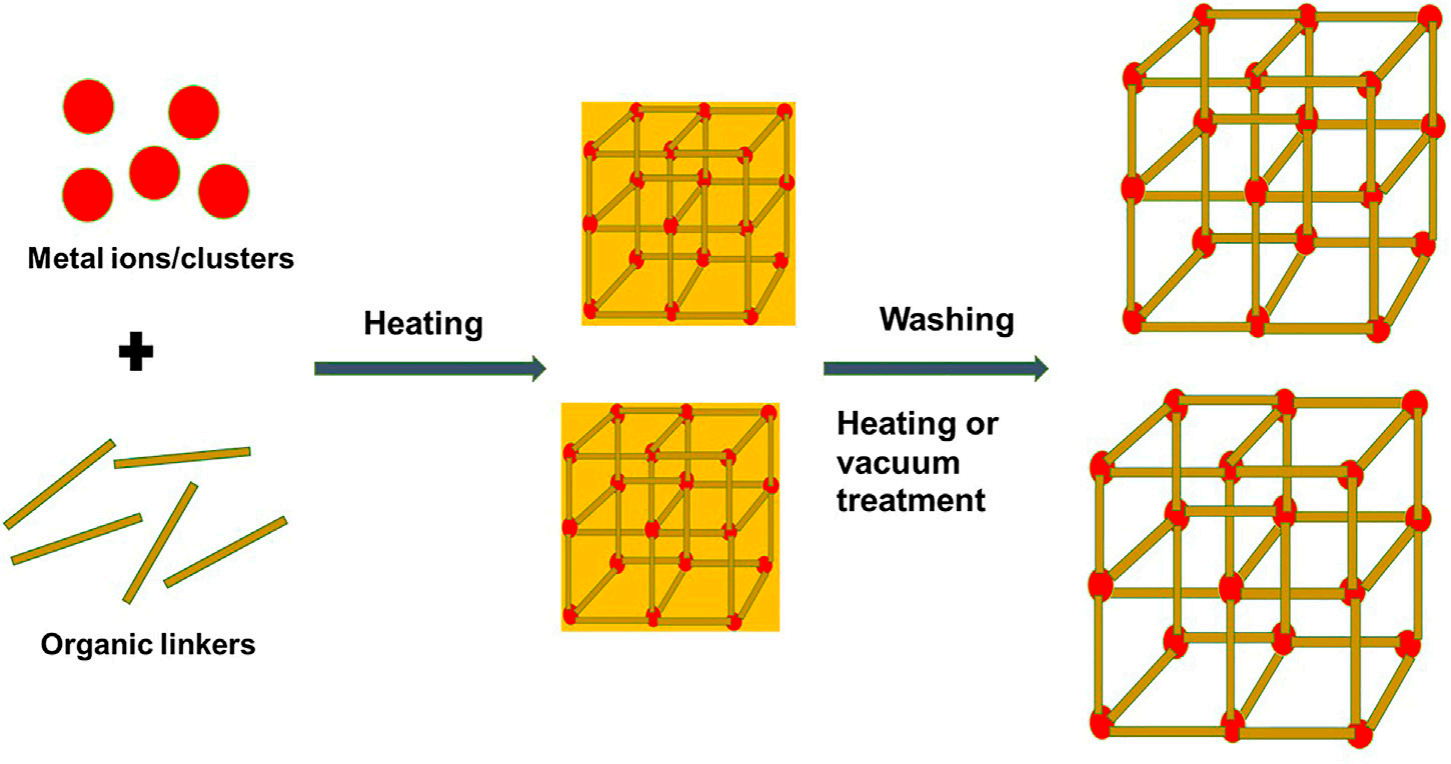
Schematic diagram of a typical component, structure of NMOFs, and synthesis using the solvothermal method as an example.

MOFs, also known as coordination networks or porous coordination polymers (PCPs), are a group of hybrid complexes with exceptional porosity formed by the self-assembly of metal-containing nodes (cations, anions, clusters, or chains) and polydentate organic ligands (imidazolates, carboxylates, or phosphonates) linked by coordination bonds that result in porous structures (Zhou et al., 2012; Furukawa et al., 2013) (Figure 2). The abbreviation “MOF” was first introduced by Yaghi et al. (1999) during their first report on an MOF structure (MOF-5), which began a new development of supramolecular structures in chemistry with a large surface area and unique nano-porosity (Yaghi et al., 1999). Ever since this development, porous hybrid structures consisting of organic and inorganic building blocks were designed and developed for various applications such as catalysis, gas storage, and drug delivery and separation (Rocca and Lin, 2011; Liu et al., 2018; Lai et al., 2019).

Recently, studies have demonstrated that MOFs scaled down to the submicron size converts to nanoscale MOFs (NMOFs) and offer unique properties related to nanomaterials (Gao et al., 2020). The combination of the two components for NMOF synthesis, the organic linker and metal ions, provides never-ending possibilities. The properties of NMOFs become a synergy of the physicochemical properties of the metal cluster with the organic material (Zhou et al., 2012). It is to be noted that mild preparation conditions and facile synthesis of NMOFs are crucially important to achieve NMOFs with specific properties and advantages for applications in drug delivery and TPDT (Lai et al., 2019). NMOFs have properties different from their bulk counterparts, such as unique properties of biodegradability, high loading ability, and specific scale size that allow for permeation through EPR and improved accumulation in the disease site (Zhu et al., 2019b). This has promoted their use as delivery vehicles of therapeutic agents (photosensitizers, target ligands, or therapy drugs) in cancer therapy (Sun et al., 2014).

### Preparations of NMOFs

Preparation methods for NMOFs include the solvothermal method, reverse microemulsion method, mechanochemical method, nanoprecipitation, electrochemical method, and post-synthetic methods (Liu et al., 2018; Zhu et al., 2019b); Table 1 shows examples of NMOFs prepared using common methods for TPDT applications. It is worth noting that reaction conditions such as the synthetic method and synthetic conditions (pH, solvent, metal–ligand ratio, reaction time, and reaction temperature) affect the structure of NMOFs and their physicochemical properties (Liu et al., 2018). Typically, nucleation and growth of NMOFs occur after precursors are mixed and allowed to react (Rocca and Lin, 2011), followed by washing or purification to achieve the designed NMOFs with porous phases (Figure 2). Some studies have demonstrated that the introduction of surfactants is able to control the size and shape of the nanocrystal during the growth stages, more so during the solvothermal and microemulsion reaction methods (He et al., 2015). Solvothermal synthesis of NMOFs involves high reaction temperatures or conventional heating to transform precursor complexes in the solvent mixture before the formation of NMOFs (He et al., 2015). In this method, the desired growth and nucleation of NMOFs occurs through control temperature and heating rates (Zhu et al., 2019b). Typically, a solution of metal ions and ligands is heated at a high temperature to undergo nucleation and growth of NMOFs and is then followed by washing or vacuum treatment to yield the desired NMOF with strong crystallinity and purity (Figure 2) (He et al., 2015; Zhu et al., 2019b). In recent studies, Fe-terephthalic acid (BDC)-based NMOFs were developed using the solvothermal method. For example, octahedral-shaped Fe_3_(µ_3_-O)Cl(H_2_O) (BDC)_3_ NMOFs with an average diameter of 200 nm were prepared by the solvothermal method through traditional heating and micro-oven heating (Horcajada et al., 2010). The Fe-BDC NMOFs displayed a highly crystalline structure that matched with the known MIL-101 structure (Horcajada et al., 2010).

**TABLE 1:** Examples of NMOFs prepared using common methods for use in TPDT or combination therapy.

Synthetic method	NMOFs	Solvent system	Properties and functions	References
Solvothermal	Fe_3_(µ_3_-O)Cl(H_2_O) (BDC)_3_ NMOFs	H_2_O	Octahedral-shaped, 200 nm, chemotherapy drug delivery	Horcajada et al. (2010)
Reverse microemulsion	Gd -BHC NMOFs	H_2_O	Block-like shape, 100 nm, multimodal contrast enhancing agents	Rieter et al. (2006)
Post-synthetic	Zr-H_2_TCPP NMOFs	DMF	Spherical-shaped particles, 30–190 nm, PS-based NMOFs with folate targeting	Park et al. (2016)
Reverse microemulsion	La-DSCP@DOPA NMOFs	Aqueous	50–150 nm, delivery of cisplatin drugs	Huxford-Phillips et al. (2013)
Solvothermal	Zr_6_(µ_3_-O)_4_(µ_3_-OH)_4_(Amino-TPDC)_6_ NMOFs	DMF	Hexagonal-plate particles, 100 nm, delivery of cisplatin drugs and siRNAs	He et al. (2014)
Solvothermal	UIO-AZB	DMF	Star-shaped particles, 10–200 nm, delivery, and release of TPZ drug and chlorin-e6	Yang et al. (2022)
Hydrothermal microemulsion	[Cu_2_(ZnTcpp)H_2_O]_*n*_ NMOFs	Aqueous	Uniform plate particles, 120 nm, PS-based NMOFs	Ma et al. (2017)
Solvothermal	Zn-TCPP NMOFs	DMF: ethanol (3:1)	2D nanosheets, nuclear imaging and chemo-photodynamic therapy	Zhu et al. (2019a)
Nanoprecipitation	Tb-DSCP NMOFs	H_2_O (methanol)	Spherical-shaped particles, 58.3 nm, delivery of cisplatin drugs	Rieter et al. (2008)
Post-synthetic method	BIO-MOF-1-MIL-101	DMF	Unusual octahedral, 200 nm, delivery of cisplatin and photosensitizers	Taylor-Pashow et al. (2009)
Solvothermal and post-synthetic method	Zr-H_2_TCPP NMOFs	DMF	Spherical-shaped particles, 58 nm diameter, antimicrobial PDT and microbial sensing	Sun et al. (2020)
Modular-assisted method	ACF@PCN-222@MnO2-PEG	DMF, followed by water	Mimetic sea cucumber-shaped, 190–300 nm, ACF and photosensitizer release and delivery	Zhou et al. (2022)
*In situ* growth method	ZiF-8@mSiO2 and DHMS	Alkaline conditions	Yolk-shell and hollow shell, 150–170 nm, MOF sonosensitizers	Pan et al. (2020)
*In-situ* growth method	Pd@MOF-525@HA	DMF	Nanocubes, 10–130 nm, deeper tissue penetration and fluorescence imaging	Duan et al. (2022)
Solvothermal	W-TBP and Bi-TBP	DMF and acetic acid	Rectangular-like nanoparticle morphology, 100 nm width and 200 nm length, CpG oligodeoxynucleotide delivery	Ni et al. (2020)
Solvent-assisted ligand exchange	UIO-PDT	DMF	Octahedral morphology, 70 nm, BODIPY delivery	Wang et al. (2016)

DMF = dimethylformamide, H_2_O = water, H_2_TCPP = unmetallated 5, 10, 15, 20-tetrakis (4- carboxyphenyl) porphyrin, TCPP = tetra (carboxyphenyl) porphyrin, ZnTcPP = zinc tetra (carboxyphenyl) porphyrin.

In the reverse microemulsion method, surfactants are normally used to regulate the nucleation of precursors and the growth kinetics of the NMOFs. Rieter W.J et al. (2006) used the reverse microemulsion method to synthesize Gd-BHC (BHC = benzene hexa-carboxylic acid) NMOFs of 25 × 50 × 100 nm in dimension by mixing two precursors of GDCl_3_ or [NMeH_3_]_2_[BDC] in water (Rieter et al., 2006). The block-like crystalline NMOFs were produced at 120°C under solvothermal conditions with a surfactant-to-water ratio used to control the morphology (Rieter et al., 2006). The same method was used to synthesize DOPA (1.2 dioleoyl-*sn*-glycero-3-phosphate) capped-NMOFs (La-DSCP @DOPA) (DSCP = di (methylammonium)) of sizes 50–150 nm for targeted delivery of cisplatin drugs (Huxford-Phillips et al., 2013). In the nanoprecipitation method of NMOF synthesis, solutions consisting of precursor complexes are mixed and allowed to undergo particle nucleation and growth under ambient conditions, followed by precipitation out of the solution with a poor solvent (He et al., 2015). This method was used to prepare NMOFs consisting of Tb^3+^ ions and anticancer prodrug *c*, *c*, *t*-Pt(NH_3_)_2_Cl_2_ (succinate)_2_ (disuccinatocisplatin, DSCP) as the organic complex. The NMOFs were prepared through nanoprecipitation by adjusting the pH of the TbCl_3_ aqueous solution and [NmeH_3_]_2_DSCP to 5.5 and precipitating the NMOF out of the solution by adding methanol (Rieter et al., 2008).

In a post-synthetic method of synthesizing or modification of NMOFs for targeted therapies, solvent-assisted ligand incorporation (SALI), solvent-assisted linker exchange (SALE), and atomic layer deposition in MOFs (AIM) are common techniques used in the post-synthetic method (Islamoglu et al., 2017). Taylor-Pashow et al. (2009) used the post-synthetic method to add therapeutic agents through covalent crafting with the carboxylate-bridging ligands of the NMOF (MIL-101 (Fe)) (Taylor-Pashow et al., 2009), while other studies used the post-synthetic method for PEGylation and addition of a porphyrin photosensitizer on the surface of the Hf-NMOF (Liu et al., 2016) and Zn-NMOF (Zhu et al., 2019a) at room temperature. A post-synthetic method (PSM) involving the addition of a photosensitizer molecule was also used by Wang et al. (2016) by using solvent-assisted ligand exchange to add a BODIPY (4, 4- difluoro-4-bora-3a, 4a-diaza-s-indacene) dye as a ligand on the UIO NMOF framework (Wang et al., 2016). The NMOF complex was stable before and after the post-synthetic procedure and was able to be used as a PDT photosensitizer on various cancer cells (Wang et al., 2016).

## NMOFs in photodynamic therapy

### NMOFs as photosensitizers

**FIGURE 3:**
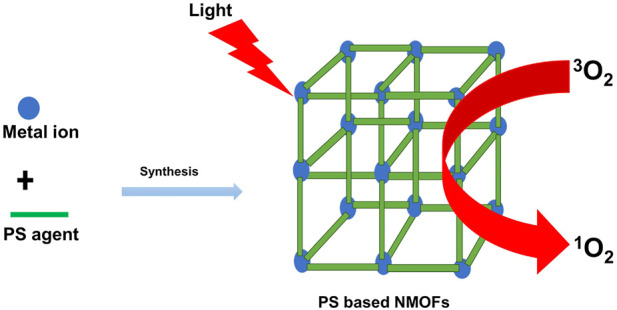
Schematic illustration of the synthesis and application of PS-based NMOFs in TPDT.

A photosensitizer (PS) agent is one of the key components in TPDT as it is the only component that can be excited to generate ROS or cytotoxic singlet oxygen during therapy (Kwiatkowski et al., 2018). PS agent-based NMOFs are prepared from commonly known organic photosensitizer complexes such as BODIPY (4,4-difluoro-4-bora-3a, 4a-diaza-s-indacene) dyes and porphyrins and their derivatives (Alves et al., 2021) as organic ligands and metal ions or clusters (Figure 3). The resulting PS agent-based NMOFs are able to absorb light to undergo photochemical pathways and yield cytotoxic singlet oxygen in the presence of molecular oxygen, causing cell death in a typical tumor microenvironment (Lim et al., 2013) (Figure 3). In PS agent-based NMOFs, the metal ions/clusters of the NMOFs are crucial in improving the efficacy of TPDT through alleviation of hypoxia, to increase the yield of cytotoxic singlet oxygen or ROS, decrease antioxidant species, and to act as contrast agent in image-guided therapy (Alves et al., 2021). The morphology and size of the NMOFs are also important in modulating the cellular uptake, the diffusion of ROS or singlet oxygen, and clearance post-treatment. An NMOF requires sizes that can easily permeate and concentrate at the tumor microenvironment through leaky vasculature systems, yet not large enough to be able to retain efficiently within the disease site (Kohane, 2007; Faraji and Wipf, 2009).

PS agent-based NMOFs were reported by various studies as listed with examples in Table 2. Porphyrin and its derivatives and BODIPY dyes are the most explored conventionally known PS agents used as organic ligands on PS agent-based NMOFs (Taylor-Pashow et al., 2009; Liu et al., 2016; Zhu et al., 2019a; Meng et al., 2021). Liu et al. (2016) prepared a PS agent-based NMOF by coordination of Hafnium (Hf^4+^) as metal nodes with tetrakis (4-carboxyphenyl) porphyrin (TCPP) as the organic ligand to form Hf-TCPP PS-agent NMOFs (Liu et al., 2016). The PS agent-NMOF showed excellent multifunctionality for combined therapy that includes PDT which led to the remarkable elimination of tumor in *in vivo*. The inherent properties of the metal ions and the photosensitizer ligand promoted excellent biodegradability and demonstrated significant potential in clinical translation (Liu et al., 2016). Similarly, Park et al. prepared a PS-based NMOF using Zr^6+^ as the metal node and TCPP as the organic ligand to form a precisely tuned size NMOF (Zr-TCPP, PCN-224) that was used for PDT on Hela and A549 cells (Park et al., 2016). The ability to have an optimal size (90 nm) PS-based NMOF was demonstrated with excellent photosensitizing properties that led to improved PDT efficacy as compared to porphyrin alone (Park et al., 2016).

**TABLE 2:** Examples of NMOFs and PS agent-based NMOFs in TPDT and their targeting properties.

NMOF	PS agent	Modification methods	Cancer cell-line or animal models	Targeting	Ref
Hf-TCPP NMOFs	Porphyrin	Nanocage, PEGylation	Hela cells, NIH3T3 cells	EPR (passive)	Liu et al. (2016)
Zn-TCPP NMOFs	Porphyrin	Dox loading, PEGylation	4T1 cells, CT26 cells, MCF7 cells	EPR (passive)	Zhu et al. (2019a)
BDC-NH-BODIPY NMOFs	BODIPY dye	Covalent modification	HT-29 cells	EPR (passive)	Taylor-Pashow et al. (2009)
Zr-H_2_TCPP NMOFs	Porphyrin	Folic acid modification	Hela cells	Active (folate targeting)	Park et al. (2016)
NP-1-ZnTcpp NMOFs	Porphyrin	PEGylation	HepG2 cells, LoVo and HCT116 nude mice	Stimuli (hydrogen sulfide (H_2_S))	Ma et al. (2017)
Poly(DH-Se/PEG/PPG urethane -PCN-224 NMOF	Porphyrin	PEGylation, Dox loading	HepG2 cells, Mice bearing HepG2	Passive (EPR) and stimuli (REDOX cleavable)	Luo et al. (2019)
Dox@PCN-224-DNA NMOFs	Porphyrin	DNA (aptamer) functionalization, Dox loading	A549 cells, MCF-7 cells	Active (aptamer targeting)	Zhang et al. (2019)
PCN-224 (Zr/TI) NMOFs	Titanium	Cation exchange	Multidrug-resistant bacteria	-	Chen et al. (2020)
Dox@BBP-MOFs	BODIPY dye	Dox loading	Hela cells	EPR (passive)	Meng et al. (2021)
Porphyrin-MnO_2_ NMOFs	Porphyrin, MnO_2_	PEGylation	CT26 cells	EPR (passive)	Zhu et al. (2021)
UiO-AZB/HC-TPZ	Chlorin e6(Ce6)	Human serum albumin (HSA), triapazamine (TPZ)	4T1 cells, 4T1 tumor-bearing nude mice	EPR (passive) and stimuli (hypoxia-activated)	Yang et al. (2022)
ACF@PCN-222@MnO2-PEG (APM)	Porphyrin	PEGylation, MnO2	Hela cells, U14 cells-bearing female Kunming mouse model	Stimuli (hypoxic, H_2_O_2_-triggered drug release)	Zhou et al. (2022)
UiO-PDT	BODIPY	-	B16F10, CT26 and C26 cells	EPR (passive)	Wang et al. (2016)
Hf-UiO-AM@POP-PEG	Tetrakis (4-aminophenyl)-21H,23H-chlorin (TAPC)	PEGylation	Hela cells, HepG2, U14 cervical cancer bearing mice	EPR (passive)	Zheng et al. (2020)
UiO-AM@BODIPY	BODIPY	-	Hela cells, L929 cells	EPR (passive), stimuli (pH responsive)	Liu et al. (2020)
PCN-222-SO3H (PCN-SU)	Porphyrin	-	4T1 cells and 4T1 breast tumor-bearing mice	EPR (passive)	Li et al. (2022)
IL@MIL-101(Fe)@BSA-AuNCs	-	-	HepG2, L929, H22 cells and H22 tumor-bearing mice	EPR (passive)	Ma et al. (2019)

PCN-224 = Zr_6_–porphyrin NMOF, MnO_2_ = manganese oxide, EPR = enhanced permeability and retention; DOX = doxorubicin; PEG = polyethylene glycol.

**FIGURE 4:**
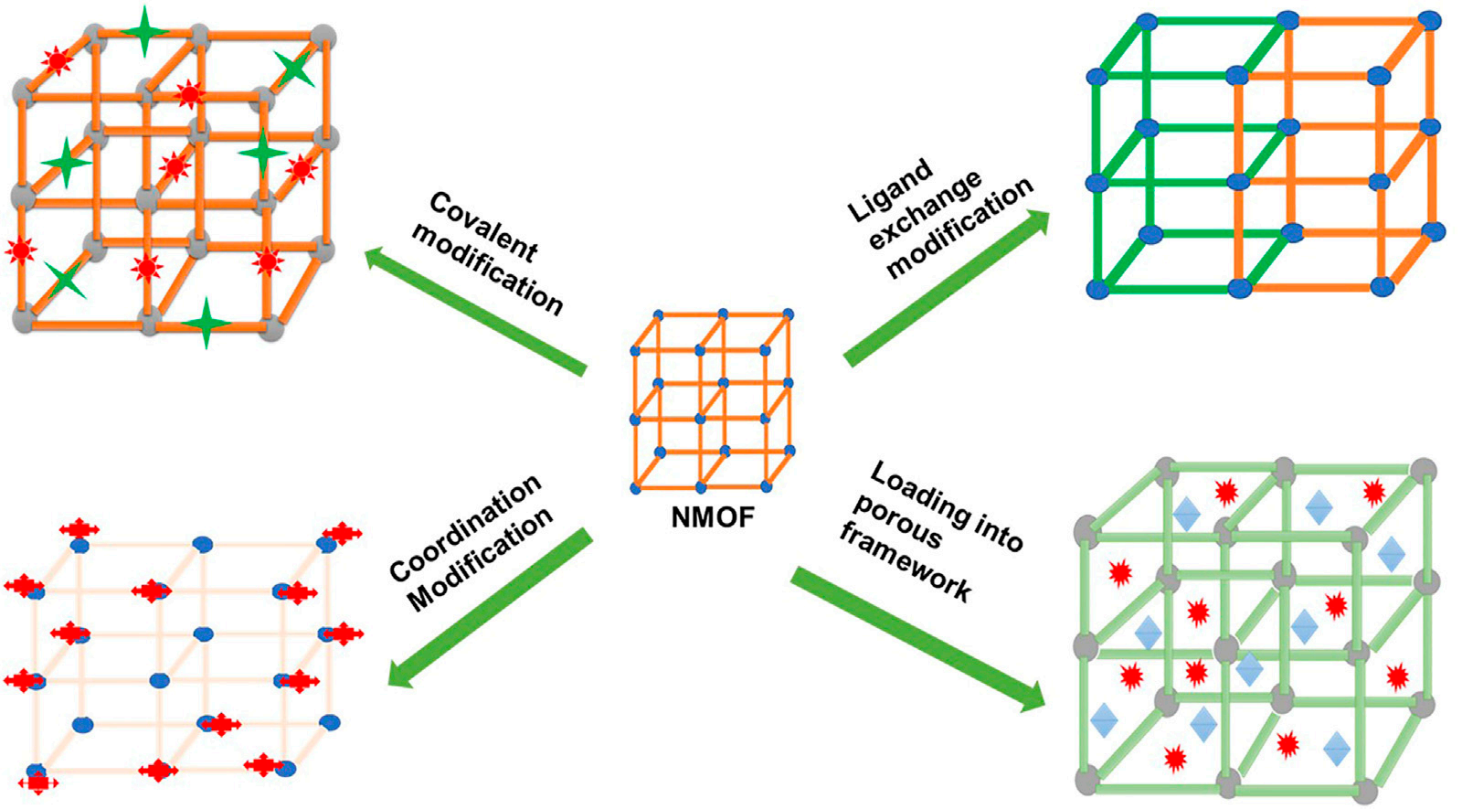
Schematic diagram depicting different ways in which the post-synthetic method can be used in NMOF modification to yield PS-based NMOFs or nanocarrier-based NMOFs.

**FIGURE 5:**
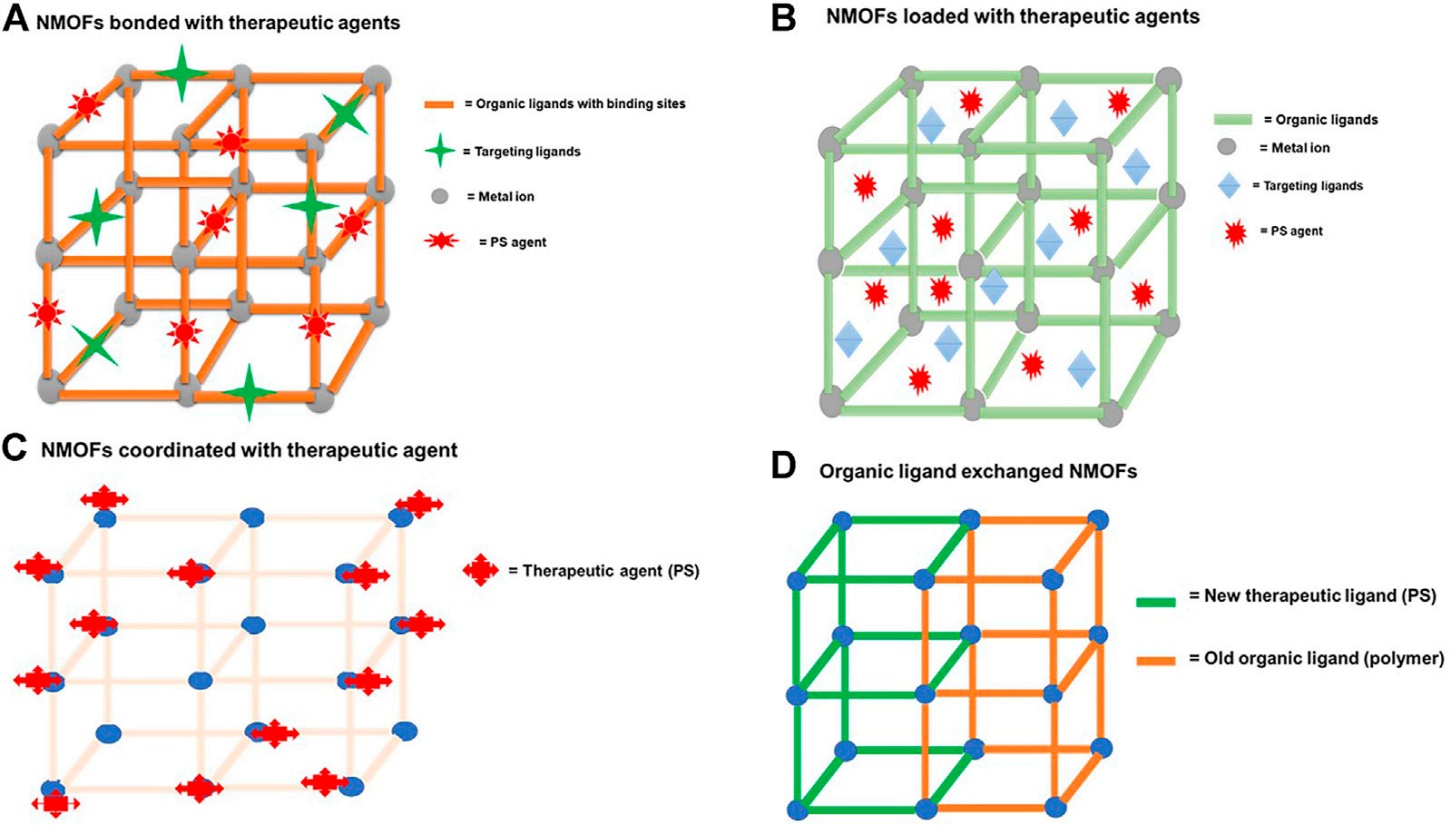
Schematic diagram depicting a typical NMOF nanocarrier **(A)** bonded through binding sites to therapeutic agents, **(B)** when therapeutic agents are incorporated or loaded in the porous matrix of the NMOFs, **(C)** when therapeutic agents (PS) are coordinated through the metal nodes, and **(D)** NMOFs’ post-ligand exchange method of the organic ligand with therapeutic agents.

PS agents can also be added to the framework through a post-synthetic procedure by coordination through the metal nodes, by covalently binding the PS agent to the organic linker, or by performing the ligand exchange with a therapeutic agent to replace the organic linker (Taylor-Pashow et al., 2009; Liu et al., 2016; Wang et al., 2016; Ye et al., 2022) (Figures 4, 5). Wang et al. (2016) prepared a PS-based NMOF by using a solvent-assisted ligand exchange method to attach a BODIPY ligand to the UIO-66 framework (Wang et al., 2016). The results demonstrated excellent biocompatibility and high singlet oxygen-generating properties that were used for PDT of cancer on B16F10, CT26, and C26 cell lines (Wang et al., 2016).

### NMOFs as nanocarriers

NMOFs consisting of metal clusters and organic ligands have emerged as a highly promising nanocarrier due to their adjustable compositions, ultrahigh specific surface area, and intrinsic biodegradability (Sun et al., 2014). NMOFs consist of a porous structure that allows the incorporation or encapsulation of therapeutic agents such as photosensitizers, chemotherapeutic drugs, target ligands, and nucleic acids (Stuchinskaya et al., 2011; Parveen et al., 2012). Figure 5 shows both an NMOF with a capacity of loading the therapeutic agents (target ligands and PS agents) through the porous matrix and through the covalent linkage or chemical bonding with the binding sites of the organic complex. NMOFs are highly porous structures, and taking advantage of this property and the functionality (binding sites) of the organic ligands, various strategies of incorporating and high loading of therapeutic agents onto or into NMOFs can be achieved (Figure 4). NMOFs as nanocarriers offer hybrid or synergistic properties of both the inorganic component and the organic component of the structure (Matlou and Abrahamse, 2021), allowing diverse properties to be exploited in applications.

Table 2 shows examples of some of the NMOFs that have been successfully designed and used by various researchers to load therapeutic agents such as DOX, PEG, aptamers, and PS agents (porphyrin, titanium, and BODIPY). Therapeutic agents were either loaded through encapsulation within the porous structure (Liu et al., 2016; Zhang et al., 2019) or by covalent binding on the organic ligand matrix (Taylor-Pashow et al., 2009) and cation exchange of central metals (Chen et al., 2020). Target ligands such as aptamers and folic acid are important to improve the active targeting of the NMOF in cancer therapy (Akhtar et al., 2014). Aptamers improve target-specific recognition and homologous accumulation abilities of the NMOF in the tumor microenvironment while folic acid is able to bind to the folic receptors that are overexpressed on the cancer surface, thus accelerating drug concentration in tumor cells (García-Díaz et al., 2011; Akhtar et al., 2014). Recently, Zhang et al. also prepared an NMOF nanocarrier by using a post-synthetic modification method to covalently attach targeting therapeutic agents (aptamer) to the PCN-224 NMOF framework (Zhang et al., 2019). The photocytotoxicity of the NMOF in this work was found to be better due to the improved targeting abilities of the aptamer ligand to A549 lung cancer cells (Zhang et al., 2019).

## Conclusion, challenges, and future perspectives

Recently, NMOFs have demonstrated great potential for applications in PDT due to their distinctive properties within their structure that include large surface area, high porosity, and tunable composition. Incorporating PS agents and other targeting therapeutic agents in NMOFs with their different modifications stand to benefit PDT treatment by overcoming its drawbacks set by previous-generation PS agents. These loaded agents create a multifunctional platform to explore synergy and combination therapy to provide complete tumor eradication with other therapies such as radiotherapy, immunotherapy, photothermal therapy, and gas-mediated therapy. These combined therapies lead to clinical benefits in treating metastatic cancer and other challenging cancer cases. The main advantage is that the metal ions add a novel, improved, and synergistic property to the NMOFs as nanocarriers or as PS agent NMOFs which also improves the photosensitizing properties of the NMOFs’ PS agents. The framework increases ROS diffusion, improves chemical and photo-stability, and advances drug delivery mechanisms and targeting.

Although the most common method of preparing NMOFs is solvothermal, the nucleation and growth mechanism of NMOFs and their crystallographic isolation are still not clear. It is, therefore, important to understand the growth and nucleation mechanism to construct NMOFs with a targeted framework, uniform and hierarchical pores, and adequate composition. Currently, the PS agent NMOFs are being explored for the use of porphyrins as their organic linker, and various other organic photoactive dyes with higher singlet oxygen-generating abilities are unexplored. NMOFs possess various advantages in TPDT because they offer both attachment and encapsulation of therapeutic agents and the rational design of PS agent-based NMOFs where a PS agent is an organic linker. In principle, NMOF-based PS agents or drug delivery systems should integrate diagnostic abilities, high drug loading capacity, long circulation times, effective targeting methods, drug release programmability, stimuli responsiveness, and effective clearance from the host body after treatment. In addition, NMOFs with imaging agents need to be considered together with various metal ions. This could also open up an opportunity of exploring NMOFs with two or more different metal nodes on one framework.

Since there are still limited clinical applications of NMOFs, it is important to consider *in vivo* clearance after treatment during the design stages of the NMOFs as carriers or as PS agents. Therefore, designing NMOFs with a capacity of targeting different tumor microenvironments, effectively destructing the cells through ROS or cytotoxic singlet oxygen cancer killing, followed by clearance from the system is vital for a proper NMOF system in clinical applications with TPDT. Stimuli-responsive properties of NMOFs also need to be considered, especially with regard to the tumor microenvironment; this could create a multifunctional platform that completely targets and releases drugs at the tumor site, which improves therapy. There is also a vast opportunity to explore other organic photoactive dyes beyond porphyrins and porphyrin derivatives in PS agent-based NMOFs. Since the bulk synthesis of NMOFs is still challenging, an efficient method with high purity and ability to scale for large use needs to be developed with the aim of applying NMOFs in biomedicine and achieving the desired selectivity with equivalent or advanced therapeutic results.
